# Analysis of lockdown perception in the United States during the COVID-19 pandemic

**DOI:** 10.1140/epjs/s11734-021-00265-z

**Published:** 2021-09-01

**Authors:** Francesco Vincenzo Surano, Maurizio Porfiri, Alessandro Rizzo

**Affiliations:** 1 Dipartimento di Elettronica e Telecomunicazioni, Politecnico di Torino, Turin, Italy; 2 Department of Mechanical and Aerospace Engineering, Tandon School of Engineering, New York University, Brooklyn, NY USA; 3 Center for Urban Science and Progress, Tandon School of Engineering, New York University, Brooklyn, NY USA; 4 Department of Biomedical Engineering, Tandon School of Engineering, New York University, Brooklyn, NY USA; 5 Office of Innovation, Tandon School of Engineering, New York University, Brooklyn, NY USA

## Abstract

Containment measures have been applied throughout the world to halt the COVID-19 pandemic. In the United States, several forms of lockdown have been adopted in different parts of the country, leading to heterogeneous epidemiological, social, and economic effects. Here, we present a spatio-temporal analysis of a Twitter dataset comprising 1.3 million geo-localized Tweets about lockdown, from January to May 2020. Through sentiment analysis, we classified Tweets as expressing positive or negative emotions about lockdown, demonstrating a change in perception during the course of the pandemic modulated by socio-economic factors. A transfer entropy analysis of the time series of Tweets unveiled that the emotions in different parts of the country did not evolve independently. Rather, they were mediated by spatial interactions, which were also related to socio-ecomomic factors and, arguably, to political orientations. This study constitutes a first, necessary step toward isolating the mechanisms underlying the acceptance of public health interventions from highly resolved online datasets.

## Introduction

The word “lockdown” originated in the context of criminal justice in the middle of the 20th century [1], indicating an emergency measure in which people are temporarily prevented from entering or leaving a restricted area. Since the first wave of SARS-CoV-2 in 2019, this word has been utilized to broadly define the measures adopted by governments and local administrations to curb the diffusion of the epidemic, by reducing individuals’ mobility and in-person interactions. These measures include restricted access to shops, workplaces, and other public spaces, along with travel limitations. With their high population densities and productive and economic fabric, cities have been dramatically affected by the pandemic and its containment measures [2]. Lockdowns have had a broad and strong impact on the life of individuals and communities [3–5], who have experienced different psychological responses that evolved over time. While such measures are undoubtedly beneficial from an epidemiological point of view, their economic, social, and psychological costs cannot be denied.

The adoption of lockdown measures to curb the diffusion of COVID-19 has impacted social interactions, accelerating massive use of online platforms at a rate even faster than the spread of the epidemic [6–8]. Among social media, Twitter is one of the preferred platforms for users to express their reactions to the ongoing epidemics and related policies [9, 10]. Twitter is a micro-blogging platform, where users can write posts of up to 280 characters, including images and URLs. Users interact through re-Tweets, by forwarding the text of others on their own post stream; mentions, where users explicitly refer to others in their Tweets; and follows, where users decide to permanently incorporate others’ Tweets in their stream.

Twitter has been studied by researchers to investigate public opinion on a variety of topics. Notably, Twitter was extensively used to understand how the political debate evolved and was perceived [11–15], investigate how rumors and opinions spread [16, 17], and test the validity of models of complex social behavior [18–20]. Other efforts aimed at understanding the spread of contagious diseases that would be otherwise hard to track with traditional medical testing [21], such as influenza [22–27], Ebola virus disease [28–30], and, more recently, COVID-19 [31].

The availability of data about COVID-19 diffusion and the access to Twitter data enabled different studies on the perception of and reaction to the pandemic [32]. Typically, these studies rely on sentiment analysis, also known as opinion mining [33]. The tools used in sentiment analysis are statistical techniques that explore and extract emotions conveyed by selected texts [34–39], in terms of a discrete classification or a continuous score. Twitter data on COVID-19 pandemic has been used to study reactions to the outbreak in different countries [40–42], benchmark and validate new models for natural language processing [43–45], perform sentiment analysis about the pandemic [46–48], and conduct analyses about a specific event [49].

Of the entire body of knowledge on the topic, only the study by Rahman et al. [49] frames the sentiment analysis within a socio-economic perspective, although relying on a relatively small dataset. Other authors have used Twitter to study real-time events [50], mostly relying on a limited number of interactions [51] or tackling the analysis mainly from a theoretical point of view [52]. To the best of our knowledge, sentiment analysis on a big dataset collected over long periods of time remains elusive, especially in the context of a disruptive event, such as the COVID-19 pandemic.

In this vein, the present study explores temporal variations in the emotions expressed online by Twitter users regarding lockdown measures in the United States (U.S.), starting from what is commonly referred to as the first wave of the virus (January–May 2020). To identify the drivers of sentiment dynamics, we consider spatio-temporal variations in the severity of the pandemic, along with social, economical, and political aspects. Within an information-theoretic approach, we use the notion of transfer entropy [53] to discover causal relationships that underlie the spread of emotional content among different geographical regions in the U.S. Toward the identification of salient factors, we then proceed to a dimensionality reduction using principal component analysis. In light of the granularity and extent of the available data, we are successful in spatially correlating emotional shifts to epidemic prevalence and socio-economic factors.

## Methods

We examined the sentiment expressed in the online debate surrounding the containment policies to combat COVID-19 in the U.S. between January 21st and May 31st 2020. The data we processed comprise about 55 million Tweets in English [32], as defined by Twitter’s metadata. The data was subsequently filtered to retain only those originating from one of the fifty U.S. states or from the District of Columbia. We performed a polar sentiment analysis [54] on all Tweets containing the word “lockdown,” categorizing them as expressions of positive, negative, or neutral emotions. For each U.S. state and the District of Columbia, we recorded the daily portion of positive and negative Tweets. Alongside these data, we collected the number of daily infections in the U.S. from the publicly available dataset of the New York Times [55], and several socio-economic indicators from the Census Bureau website [56].

### Data, pre-processing, and post-processing

Our analysis is based on the ongoing collection of data curated by Chen et al. [32], which started on January 21st, 2020, and which included more than 123 million Tweets in several languages when this project started. To comply with the Twitter privacy policy, the database contains only Tweets IDs. We used the software Hydrator [57] to retrieve the Tweets text and metadata. Specifically, metadata are used to select only Tweets written in English. Re-tweets are not distinguished from ordinary Tweets, under the premise that a user who re-Tweets is expressing a form of endorsement [36].

We filtered the data set by restricting the search to Tweets containing the keywords established by the data set curator before February 16th 2020. Specifically, we used the following keywords: “Coronavirus”, “Corona”, “CDC”, “Ncov”, “Wuhan”, “Outbreak”, “China”, “Koronavirus”, “Wuhancoronavirus”, “Wuhanlockdown”, “N95”, “Kungflu”, “Epidemic”, “Sinophobia”, and “Covid-19”. Starting from such a filtered data set, we restricted our field of analysis to those Tweets containing the term “lockdown,” either as Tweet text or as a hashtag, regardless of any capitalization. Only Tweets originated in the U.S. have been retained, through a geo-localization procedure detailed in what follows. Eventually, the data set contained about 1.3 million Tweets, monthly distributed as follows: January, 56, 920; February, 40, 030; March, 322, 877; April, 857, 612; and May, 32, 865.

Multiple metadata are associated with Tweets, thereby allowing for inferring the position of the user at the time of content creation or their home and workplace. The largest portion of Tweets, ranging from $99.69\%$ to $99.92\%$, have a user-defined location. This is likely connected to users’ home or workplace [58], although it may not reflect their exact position and, sometimes, does not contain meaningful information (referring, for example, to imaginary places, or to whole countries [58]). A much smaller portion of Tweets is associated with platform-generated locations, based on the Tweet content ($0.11\%-0.26\%$). An even smaller portion of Tweets contains a GPS location ($0.02\%-0.08\%$).

To associate specific coordinates to each Tweet we relied on the geoparsing software CLIFF-CLAVIN [59]. Upon retrieval of a geographical entity in the Tweet, we used the open data provided by OpenStreetMap Contributors$\copyright$  to determine the country of origin. If the Tweet is originated in the U.S., we sought to narrow the origin to any of the fifty states or the District of Columbia. In case of conflicting information regarding the state of origin, we discarded the Tweet.

We studied polarization and changes in sentiment in the online debate about the topic of lockdown using a classification of emotions aroused by text, in positive, neutral, or negative. Such an analysis was performed using VADER [54], a valence-aware sentiment analysis tool. For each Tweet, VADER assigns a composite score that is used for classification. Specifically, following [54], we selected three thresholds to assign an emotional quality to each Tweet. Composite scores below $-0.050$ were classified as carrying negative emotions; between $-0.050$ and 0.050 as neutral; and above 0.050 as carrying positive emotions.

By performing sentiment analysis on the geo-localized Tweets, we created two local time-series for each region (all the U.S. states and the District of Columbia), namely, daily fractions of positive Tweets $\rho _P(t)$ and negative Tweets, $\rho _N(t)$. In total, we collected 102 local time-series, with the resolution of one day, each one with a length of 132 days.

To acknowledge country-wise changes in the perception of the pandemic, we partitioned each time-series (from the fifty U.S. states and the District of Columbia) in three sections: before the onset of the pandemic (the first day in which the incidence of 5/10, 000, 000 daily cases in the population of the corresponding region was registered), from the onset of the pandemic to the first peak of the infection incidence (evaluated using a moving weekly average), and from such a peak to the end of May 2020.

For each region, we studied the time-series of the portion of positive and negative Tweets over the total number of Tweets, $\rho _{P} (t)$ and $\rho _{N} (t)$. From each of these time-series, we computed the average values over the three sections, $\rho _{P}^i$ and $\rho _{N}^i$, and the standard deviations, $\sigma _{P}^i$ and $\sigma _{N}^i$, with $i=\{1,2,3\}$. To ascertain time variations in the positive and negative sentiments across the three sections, we used a Welch’s *t*-test with a significance level of 0.050.

### Socio-economic factors

We considered education and wealth indicators from the 2018 data of the U.S. Census Bureau [56]. For each region (U.S. state or the District of Columbia), we collected the corresponding data for Population (*POP*), Median Household Income (*MHI*), and the following rates: Poverty (*PR*), Employment (*ER*), Uninsured (*UR*), High School Diploma (or higher level, *HSD*), Bachelor Degree (*BD*), and Professional or Doctoral Degree (*PDD*).

To consolidate the number of explanatory variables into interpretable indicators [60], we performed a principal component analysis on these socio-economic factors [61]. We retained three main components, accounting for $73\%$ of the total variance and all having a corresponding eigenvalue above 0.995. We excluded variables contributing to a principal component with an absolute loading lower than 0.500. The first principal component, accounting for $37\%$ of the variance, is interpreted as “Wealth” and is mainly associated with the poverty rate (principal component loading equal to $-0.958$), employment rate (0.816), rate of Bachelor Degree (0.768), and median household income (0.673). The second principal component, accounting for $27\%$ of the variance, is interpreted as “Education” and is mainly associated with the rate of Professional or Doctoral Degree (loading equal to 0.940), the Median Household Income (0.599), the rate of Bachelor Degree (0.557), and the rate of High School Diplomas ($-0.523$). Finally, the third principal component, accounting for $10\%$ of the variance, is interpreted as “Social Exclusion” and is mainly associated with the rate of high school degree ($-0.562$) and the rate of uninsured (loading equal to 0.553).

The obtained principal component scores were used as dependent variables in a Kendall correlation test [62] with combinations of sentiment analysis parameters. The null-hypothesis of independence was tested with a two-sided test with $p<0.050$.

### Spatial interactions

Given the massive use of Twitter throughout the country, it is tenable to expect that local sentiment does not evolve in silos, but is the result of a spatial influence process. Hence, we studied the influence of sentiments among regions. We pursued this analysis through an information-theoretic approach based on the notion of transfer entropy. Transfer entropy is designed to unveil cause-and-effect relationships in a Wiener-Granger sense. Specifically, a process *X* is said to cause another process *Y* if knowledge of the present state of *X* improves the prediction of the future of *Y* from its present [53].

We separately studied spatial interactions associated with positive and negative Tweets. For each type of Tweet, we computed transfer entropy between any pair of local time-series, totaling $51 \times 50 = 2,550$ values of transfer entropy. To control for common-driver effects in the evolution of time-series (for example, one state simultaneously influencing two other states that would otherwise be independent), we conditioned over the average of positive or negative Tweets across the entire country. Specifically, given a source process *X* (local time-series of positive or negative Tweets), a target process *Y* (local time-series of positive or negative Tweets), and the conditioning process *Z* (national average of time-series of positive or negative Tweets), we computed conditional transfer entropy as

1 $$\begin{aligned} TE_{X\rightarrow Y|Z}= & {} H(Y(t+1)|Y(t),Z(t))\nonumber \\&-H(Y(t+1)|Y(t),X(t),Z(t)), \end{aligned}$$

where $H(\cdot )$ is the Shannon entropy.

In the computation of transfer entropy, we used a symbolic representation with a binary alphabet to ensure the accuracy of the estimation of the probability mass functions in the Shannon entropy, similar to our previous work [63]. Specifically, we first detrended the local time-series of positive and negative Tweets by subtracting at each instant of time the average value of the corresponding time section (before the onset of the pandemic, from the onset of the pandemic to the incidence peak, from the incidence peak to the end of May 2020); we verified the stationarity of the time-series using a Dickey-Fuller test [64]. Then, we symbolized the time series into a sequence of binary symbols: $\uparrow$ and $\downarrow$, associated with daily values above or below the median, respectively. This transformation was performed separately for both the time-series of positive and negative Tweets, obtaining a total of 102 symbolic time-series.

Statistical testing was performed by following the approach presented in [65]. To test whether transfer entropy in Eq. (1) was different from chance, we created a surrogate distribution by shuffling the values of the source process, while preserving the associations between the target and conditional processes. A total of 10, 000 permutations were executed for each statistical test and a significance level of 0.050 was considered.

Hence, for every pair of candidate target and source processes, we rejected (or failed to reject) the null hypothesis that their directional interaction from positive or negative Tweets was due to chance. Through this analysis, we determined two directed networks, one from spatial influences inferred from positive Tweets, and the other from negative Tweets, in which a link signifies rejection of the null hypothesis. No assumption was made on the topology of these networks, so that, in principle, links between regions may emerge independently of their geographic location.

To highlight the strongest patterns of spatial influence, we studied the normalized in-degree centrality, $K_{(N,P), in}$ and the normalized out-degree centrality, $K_{(N,P), out}$ [66] of the obtained networks. The in-degree centrality of a node of a directed network is equal to the total number of links that terminate at the node, thereby measuring the extent to which the node is influenced by the rest of the network. On the contrary, the out-degree centrality is equal to the total number of links that originates at the node, thereby quantifying the overall influence of the node on the rest of the network. Both quantities were normalized by their maximum value, so that they range between zero and one.

Using the directed networks and the centralities described above, we investigated potential associations between socio-economic factors and spatial influence patterns through Kendall-$\tau$ correlation tests using a two-sided significance threshold of $p<0.050$. In addition, we sought to connect these patterns to political ideology, as defined by Berry et al. [67] and using updated 2018 data from Professor R.C. Fording [68]. To this aim, we assigned to each region a label, either “liberal” or “conservative”, and then we counted in any of the two networks the number of links connecting nodes with the same or different ideology.

## Results

**Fig. 1:**
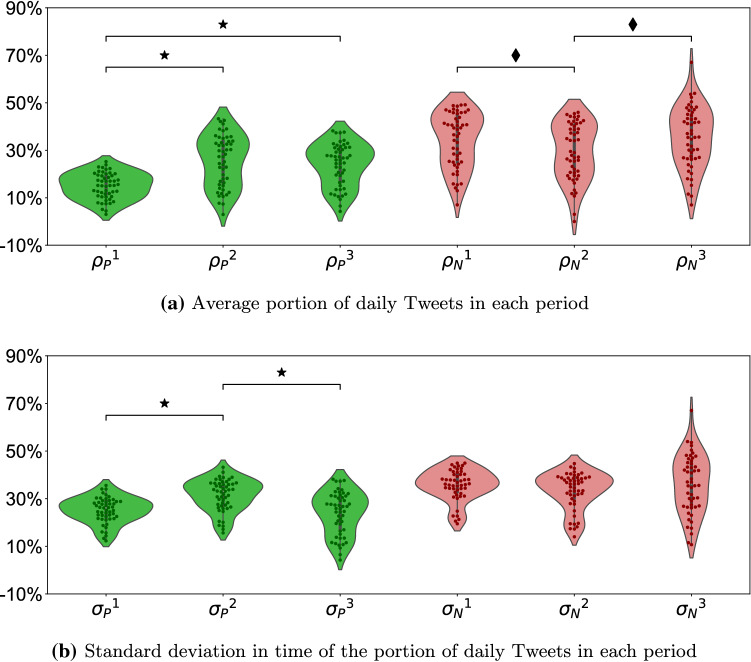
Green and red violin plots represent Tweets corresponding to positive and negative sentiments, respectively. Each point represents the value for any of the fifty U.S. states or the District of Columbia. Stars indicate significant comparisons at $p<0.001$ and diamonds at $p<0.050$

Across time, we registered a variation in both the means of the positive and negative Tweets (Fig. 1a). Specifically, the portion of positive Tweets before the onset of the pandemic was lower than the section between the onset of the pandemic and the incidence peak ($t_{74.33}=6.24$, $p<0.001$) and than the section from the incidence peak to the end of May 2020 ($t_{83.12}=6.12$, $p<0.001$). We did not register a difference between the portion of positive Tweets from the central section to the last section ($t_{96.56}=0.82$, $p=0.416$). Likewise, we determined a temporal variation in the portion of negative Tweets, whereby the central section was higher than the initial one ($t_{99.88}=2.04$, $p=0.045$) and the last section was higher than the central section ($t_{99.67}=2.44$, $p=0.016$). However, such differences did not reverberate into a significant change from the first to the last section ($t_{99.18}=0.50$, $p=0.620$).

Differences in the mean of the portion of positive Tweets in time were accompanied by changes in their variability (Fig. 1b). Specifically, the standard deviation showed an inverted U-shape, by increasing from the first to the second section ($t_{96.16}=5.26$, $p<0.001$) and decreasing from the second to the third section ($t_{91.78}=4.81$, $p<0.001$); no difference was registered when comparing the first with the last section ($t_{81.86}=0.84$, $p=0.405$). On the other hand, the variability of the portion of negative Tweets was indistinguishable in time (first versus second section: $t_{95.06}=1.66$, $p=0.101$; second versus third section: $t_{83.18}=1.27$, $p=0.207$; and first versus third section: $t_{74.82}=0.11$, $p=0.910$).

We further investigated the correlation between socio-economic factors and the shift in sentiment across the three-time sections (Table 1). The variation in the portion of positive Tweets before the onset of the pandemic and between the onset of the pandemic and the incidence peak correlates with all the identified socio-economic factors: negatively with Wealth ($\tau =-0.442$, $p<0.001$), and positively with Education and Social Exclusion ($\tau =0.500$, $p<0.001$; $\tau =0.487$, $p<0.001$; respectively). We did not observe a correlation when examining the variation in the portion of positive Tweets between the onset and the peak and after the peak with neither Wealth ($\tau =0.183$, $p=0.058$) nor Social Exclusion ($\tau =-0.228$, $p=0.270$). On the other hand, we recorded a correlation with Education ($\tau =-0.235$, $p=0.015$). Exploring the correlation between socio-economic factors and the variation in the portion of negative Tweets, we did not find a correlation between the variation from the first to the second time sections and Wealth ($\tau =-0.112$, $p=0.245$), Education ($\tau =-0.079$, $p=0.412$) or Social Exclusion ($\tau =0.082$, $p=0.393$). Likewise, we did not register a correlation between the variation in negative Tweets between the second and the third time sections and Wealth ($\tau =0.106$, $p=0.273$), Education ($\tau =-0.101$, $p=0.295$), or Social Exclusion ($\tau =-0.107$, $p=0.266$).

Not only were socio-economic factors associated with the averages of the portions of Tweets, but also were they related to the standard deviations in time of the portions of Tweets (Table 1). Across the first and second time sections, we did not register a correlation of the change of the standard deviation of positive Tweets with Wealth ($\tau =0.082$, $p=0.394$), Education ($\tau =-0.049$, $p=0.609$) or Social Exclusion ($\tau =-0.059$, $p=0.542$). Differently, such a correlation for the same data is observed between the second and the third time sections, namely, negatively with Wealth ($\tau =-0.536$, $p<0.001$) and positively with both Education ($\tau =0.550$, $p<0.001$) and Social Exclusion ($\tau =0.540$, $p<0.001$). The variation in standard deviation of the portion of negative Tweets between the first and the second time sections did not correlate with Wealth ($\tau =0.061$, $p=0.530$), Education ($\tau =-0.086$, $p=0.380$), or Social exclusion ($\tau =-0.086$, $p=0.380$). With respect to the standard deviation in the portion of negative Tweets between the second and the third time sections, we registered a negative correlation with Wealth ($\tau =-0.528$, $p<0.001$), and a positive correlation with both Education ($\tau =0.556$, $p<0.001$) and Social Exclusion ($\tau =0.543$, $p<0.001$).

**Table 1:** Kendall-$\tau$ coefficients for the correlation between socio-economic factors and changes in the averages and standard deviations of the portions of positive and negative Tweets

Kendall-$\tau$	Wealth	Education	Social Exclusion
$\rho _{P}^2-\rho _{P}^1$	**– 0.442**	**0.500**	**0.487**
	($p<0.001$)	($p<0.001$)	($p<0.001$)
$\rho _{P}^3-\rho _{P}^2$	0.183	**– 0.235**	$- 0.228$
	($p=0.058$)	($p=0.015$)	($p=0.270$)
$\rho _{N}^2-\rho _{N}^1$	$-0.112$	0.079	0.082
	($p=0.245$)	($p=0.412$)	($p=0.393$)
$\rho _{N}^3-\rho _{N}^2$	0.106	$-0.101$	$- 0.107$
	($p=0.273$)	($p=0.295$)	($p=0.266$)
$\sigma _{P}^2 - \sigma _{P}^1$	0.082	$- 0.049$	$-0.059$
	($p=0.394$)	($p=0.609$)	($p=0.542$)
$\sigma _{P}^3 - \sigma _{P}^2$	**– 0.536**	**0.550**	**0.540**
	($p<0.001$)	($p<0.001$)	($p<0.001$)
$\sigma _{N}^2 - \sigma _{N}^1$	0.061	$- 0.086$	$- 0.086$
	($p=0.530$)	($p=0.380$)	($p=0.380$)
$\sigma _{N}^3 - \sigma _{N}^2$	**– 0.528**	**0.556**	**0.543**
	($p<0.001$)	($p<0.001$)	($p<0.001$)

Numbers in parentheses report the *p*-value from the correlation; a bold value indicates $p<0.050$

**Fig. 2:**
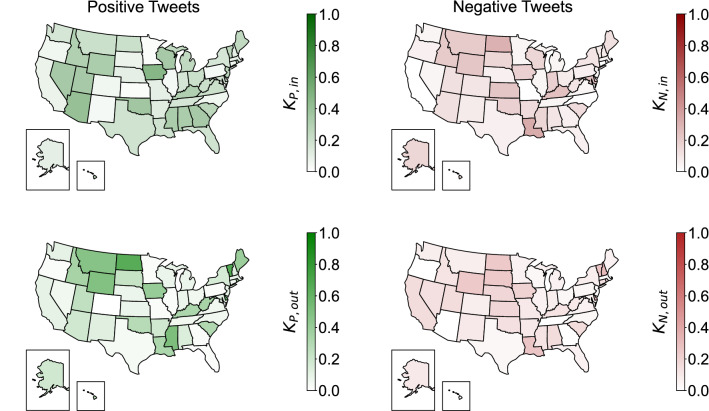
Maps of the U.S. showing the in-degree (top) and out-degree (bottom) distributions associated with the networks for positive (green) and negative (red) Tweets

In Fig. 2, we illustrate a cartographic map obtained form the transfer entropy analysis. Therein, each state is colored based on the in-degree (top images) and out-degree (bottom images) centrality as computed from the time-series of positive (green) and negative (red) Tweets: the higher the out-degree (in-degree), the higher the influence exerted (experienced) by a node on (from) the rest of the network. In total, the network of positive Tweets has 249 directed edges, whereas the network of negative Tweets contains 146 directed edges.

The in-degrees of each region, computed from the network of positive Tweets, correlate negatively with Wealth ($\tau =-0.442, p<0.001$) and positively with Education and Social Exclusion ($\tau =-0.442, p<0.001$; $\tau =-0.442, p<0.001$; respectively). On the other hand, the out-degrees computed from the same network do not correlate with any of the socio-economic factors, let them be Wealth ($\tau =0.087, p=0.383$), Education ($\tau =-0.099, p=0.322$), or Social Exclusion ($\tau =-0.102, p=0.306$). The same analysis was performed on the centrality measures for the network of negative Tweets. Here, we recorded a positive correlation between the out-degree and Wealth ($\tau =0.428, p<0.001$), and a negative correlation with Education and Social Exclusion ($\tau =-0.437, p<0.001$; $\tau =-0.440, p<0.001$; respectively). A similar pattern was noted for the in-degree, which also entailed a positive correlation with Wealth ($\tau =0.313, p=0.002$) and a negative correlation with Education and Social Exclusion ($\tau =-0.341, p<0.001$; $\tau =-0.342, p<0.001$; respectively).

**Table 2:** Kendall-$\tau$ coefficients between socio-economic factors and either in- or out-degrees from the portions of positive and negative Tweets

Kendall-$\tau$	Wealth	Education	Social Exclusion
$K_{P, out}$	**0.468**	**– 0.525**	**– 0.517**
	($p<0.001$)	($p<0.001$)	($p<0.001$)
$K_{P, in}$	0.087	$-0.099$	$-0.102$
	($p=0.383$)	($p=0.322$)	($p=0.306$)
$K_{N, out}$	**0.428**	**– 0.437**	**– 0.440**
	($p<0.001$)	($p<0.001$)	($p<0.001$)
$K_{N, in}$	**0.313**	**– 0.341**	**– 0.342**
	($p=0.002$)	($p<0.001$)	($p<0.001$)

Numbers in parentheses report the *p*-value from the correlation; a bold value indicates $p<0.050$

Finally, we performed a cluster analysis on the networks based on the liberal or conservative ideologies of the corresponding nodes. For the network associated with the positive Tweets, out of the existing 249 edges, we determined 87 ($34.9\%$) links from conservative to liberal, 76 ($30.5\%$) from to conservative to conservative, 46 ($18.5\%$) from liberal to conservative, and 40 ($16.1\%$) from liberal to liberal. For the network related to negative Tweets, out of the 146 edges, 34 ($23.3\%$) were from conservative to liberal nodes, 48 ($32.9\%$) from conservative to conservative, 37 ($25.3\%$) from liberal to conservative, and 27 ($18.5\%$) from liberal to liberal.

## Discussion

The first wave of SARS-CoV-2 has impacted the health, the wealth, and the life of millions of people all over the country. Information about the pandemic has spread over the globe, creating waves of polarized emotions and, at times, influencing actions in response to the ongoing crisis. A controversial debate has emerged about the application of strict containment policies, such as severe lockdowns and travel bans. Opinions have been extremely heterogeneous across geographical regions and social strata [69].

Here, we analyzed online sentiment on Twitter from January 21st to May 31st, 2020 in the U.S. about lockdown measures. Beyond qualitatively describing the opinion throughout the country, we sought to dissect potential explanations and causal mechanisms. In this vein, we pursued a principal component analysis on socio-economic factors to consolidate variations across the country in a few salient explanatory variables (Wealth, Education, and Social Exclusion). Alongside, we conducted a transfer entropy study to unveil spatial interactions among different regions of the country (U.S. states and the District of Columbia).

In agreement with our expectations, we registered a time variation of public opinion regarding lockdown measures. People expressed support of lockdown measures in the early stage of the pandemic, whereby the portion of positive Tweets increased and the portion of negative Tweets decreased. It is likely that risk perception regarding the spreading of the infection caused fear in the population, spurring emotional changes toward containment measures that were evident from our Twitter dataset. As the pandemic progressed, the portion of positive Tweets remained leveled and that of the negative Tweets raised, suggesting that pandemic fatigue, stress, and isolation started taking a toll [70] in how people felt about lockdowns.

Interestingly, the U.S. did not react uniformly, so that different parts of the country responded differently to the pandemic as a function of socio-economic factors. In the initial stage of the pandemic, lower Wealth and higher Education and Social Exclusion contributed to the raise in positive emotions around lockdown policies. Educated individuals, but also those fearing for their health due poverty and lack of social safety nets, were more favorable to containment measures.

As the pandemic progressed and people changed their views regarding lockdowns, these correlations were lost and, sometimes, even reversed. In particular, neither Wealth nor Social Exclusion were explanatory of the changes in positive emotions regarding lockdown. Education became negatively correlated with the sentiment change, so that people living in more affluent regions with a higher portion of college graduates were those who reduced the most their support to lockdown measures. Perhaps, this reflected some sort of cheering for the end of restrictions or the final acceptance of the new normalcy by those individuals who kept abreast of advancements about the combat against the pandemic. We warn care when interpreting this claim, whereby its statistical significance was drastically lower than any other of the observed associations and higher education was also positively correlated with changes in the temporal variability of positive sentiments, registered in our Twitter dataset and echoed by online debates [71]. As a result, claims drawn on changes in the mean values may not be indicative of a true change in sentiment.

It is tenable that the complex response of the U.S. to lockdown was mediated by spatial interactions supporting the spread of opinions across state borders. Our transfer entropy analysis offers evidence in this direction, whereby we detected close to four hundred dyadic interactions in relation to positive and negative Tweets. In agreement with one’s expectation, the distribution of these links was not at random, but rather it was informed by socio-economic factors. People living in regions with a higher Wealth tended to have a higher influence on how the rest of the country perceived lockdowns, whether through positive or negative emotions. Such an influence was, instead, moderated by Education and Social Exclusion, which may exacerbate political and cultural polarization as well as differences in the very use of Twitter [72, 73].

Interestingly, we discovered that these associations would also underlie the tendency of a region to be influenced by, rather than influence, others with respect to negative emotions. Negative emotions are likely to resonate more in wealthier parts of the country, which could have been more worried for the downturn caused by the pandemic [74]. Such a worry was indeed mitigated by higher levels of education and the presence of social safety nets. Perhaps, political orientations could play a role on these spatial interactions, but present evidence is not conclusive. We speculate that the positions on lockdowns taken by the two major parties were partly responsible for the observed spatial interactions, with conservative states playing a more influential role on opinion spreading.

Our study is not free of limitations. First, not everybody uses Twitter, so that the Twitter database may be skewed toward a fraction of the population with limited representativeness [75]. Second, we acknowledge that the Twitter database could be excessively widespread [76], thereby challenging the retrieval of pertinent information from selected keywords, especially when dealing with a new topic. Third, sentiment analysis may not allow for a deeper understanding of nuances or sarcasm [54], thereby confounding the classification of some of the Tweets in a database. Fourth, the use of aggregated socio-economic data only allows for the study of macroscopic phenomena without capturing fine details of human behavior.

There are several routes for future inquiry from this effort. In principle, our analysis could be expanded to encompass different sentiment analysis of Tweets than a simple positive/negative classification, at the cost of a more intricate interpretation of results. Likewise, our correlation studies could be undertaken without the use of a principal component analysis on socio-economic factors, thereby allowing for a more detailed assessment of potential drivers. Further work could also address a finer resolution of time effects, rather than the coarse three-section representation proposed in this work. The use of a finer resolution may help elucidate sentiment dynamics in the online debate, potentially assisting in the inference of key attributes of Tweets that become viral. Further insight could be gathered by developing a mathematical model for the dynamic evolution of the sentiment; linear spatio-temporal models could be pursued to address this need, but it is presently unclear whether the observed interactions among the regions obey to linear dependencies [77]. Although our focus was the ongoing pandemic, the approach presented in this effort could be beneficial to policy-makers when dealing with unpopular, yet timely, interventions in general [69].

## Data Availability

This manuscript has associated data in a data repository. [Authors’ comment: The data used in this work is publicly available from cited sources and repositories in references [32, 55, 56, 68].]
